# UV-B Radiation Tolerance and Temperature-Dependent Activity Within the Entomopathogenic Fungal Genus *Metarhizium* in Brazil

**DOI:** 10.3389/ffunb.2021.645737

**Published:** 2021-03-08

**Authors:** Joel da Cruz Couceiro, Maíra Blumer Fatoretto, Clarice Garcia Borges Demétrio, Nicolai Vitt Meyling, Ítalo Delalibera

**Affiliations:** ^1^Laboratory of Pathology and Microbial Control of Insects, Department of Entomology and Acarology, “Luiz de Queiroz” College of Agriculture, University of São Paulo (ESALQ/USP), Piracicaba, Brazil; ^2^Section for Organismal Biology, Department of Plant and Environmental Sciences, University of Copenhagen, Copenhagen, Denmark; ^3^Department of Exact Sciences, “Luiz de Queiroz” College of Agriculture, University of São Paulo (ESALQ/USP), Piracicaba, Brazil

**Keywords:** entomopathogenic fungus, *Metarhizium*, UV-B radiation, temperature, tolerance

## Abstract

*Metarhizium* comprises a phylogenetically diverse genus of entomopathogenic fungi. In Brazil, *Metarhizium anisopliae* s.str. subclade Mani 2 is predominantly isolated from insects, while *M. robertsii* and *M. brunneum* mostly occur in the soil environment. Solar radiation and high temperatures are important abiotic factors that can be detrimental to fungal propagules. We hypothesized that among 12 Brazilian isolates of *Metarhizium* spp., *M. anisopliae* Mani 2 (n = 6), being adapted to abiotic conditions of the phylloplane, is more tolerant to UV light and high temperatures than *M. robertsii* (n = 3) and *M. brunneum* (n = 3). Inoculum of each isolate was exposed to UV-B for up to 8 h and viability evaluated 48 h later. After 8 h under UV-B, most of the isolates had germination rates below 5%. Discs of mycelia were incubated at different temperatures, and diameter of colonies were recorded for 12 days. Mycelia of *M. robertsii* isolates grew faster at 33 °C, while *M. anisopliae* and *M. brunneum* grew most at 25 °C. Dry conidia were incubated at 20, 25 or 40 °C for 12 days, and then viabilities were examined. At 40 °C, conidia of five *M. anisopliae* isolates were the most tolerant. In the three experiments, considerable intra- and inter-specific variability was detected. The results indicate that conclusions about tolerance to these abiotic factors should be made only at the isolate level.

## Introduction

Several species within the fungal entomopathogenic genus *Metarhizium* Sorokin (Ascomycota: Hypocreales) occur worldwide in soils of natural and agricultural ecosystems (Jaronski, 2007; Lacey et al., 2015). *Metarhizium* is one of the main genera causing disease in insects (Hesketh et al., 2010). Mainly known for their action against insects, some species of this genus are also able to associate intimately with different plants as endophytes (Hu and St. Leger, 2002; Bruck, 2005; Wyrebek et al., 2011), in a relationship where the fungus can supply their plant hosts with nitrogen derived from insects (Behie et al., 2012, 2017; Behie and Bidochka, 2014; Barelli et al., 2016) as well as promote plant growth (Jaber and Enkerli, 2016a,b; Sasan and Bidochka, 2012).

In agroecosystems, *Metarhizium* communities are diverse and relatively complex (e.g., Steinwender et al., 2014), and the different species exhibit patterns of spatial distribution. In Brazil, a large diversity of *Metarhizium* spp. has been documented in soils of both natural and agricultural habitats (Rezende et al., 2015; Botelho et al., 2019). Still, there is currently limited knowledge of the characteristics of the isolates of these species of Brazilian origin.

In soils of the Cerrado biome in Brazil, Rocha et al. (2013) found a great abundance of *M. anisopliae* s.l., and by sequencing the 5′ EF-1α region of isolates from many areas of Brazil, Rezende et al. (2015) reported *M. anisopliae* s.str. haplotypes grouped in the subclades Mani 1, Mani 2, and Mani 3. *Metarhizium robertsii* Bischoff, Rehner & Humber is the most widespread and abundant species in Brazil, easily isolated from soils of many biomes (Botelho et al., 2019), while in temperate regions such as Denmark, *Metarhizium robertsii* has been recovered in low abundance in an experimental agricultural field (Steinwender et al., 2014, 2015). In contrast, *Metarhizium brunneum* Petch was recovered as the most prevalent species in agricultural soils in Denmark, while it has a limited occurrence in Brazil (Steinwender et al., 2014; Kepler et al., 2015; Brunner-Mendoza et al., 2019).

Even though the three species, *Metarhizium robertsii, Metarhizium brunneum*, and *Metarhizium anisopliae*, seemingly occupy overlapping niches, acting as entomopathogens and being found in soils worldwide (Lacey et al., 2015), there is a notable ecological difference between their distribution in Brazil. Generally, only isolates of *M. anisopliae* subclade Mani 2 are obtained as natural infections of insects collected in field crops in Brazil (Rezende et al., 2015), evidencing that members of this subclade are predominantly adapted to explore insects as a resource. On the other hand, isolates of *M. robertsii* can infect and kill different taxa of insects in laboratory conditions, but this is rarely observed in field conditions in Brazil (Lopes et al., 2013). Frequently, *M. robertsii* establishes associations with plant roots, indicating that this species is well-adapted to the soil and rhizosphere environment (Sasan and Bidochka, 2012; Rezende et al., 2015). Similarly, *M. brunneum* occurs predominantly in soils and is associated with roots (Steinwender et al., 2014, 2015), and at least in temperate regions, it is generally not found infecting insects above ground naturally (Meyling et al., 2011).

*Metarhizium* spp. are commonly found in cultivated areas and are therefore considered to be adapted to agricultural habitats (Meyling and Eilenberg, 2007; Vega et al., 2012). Abiotic factors (e.g., solar radiation, temperature, humidity, wind) can significantly influence the development, survival, and distribution of entomopathogenic fungi in the environment (Inglis et al., 2001). Agricultural habitats have limited canopy cover and are therefore considered frequently exposed to relatively high solar radiation and temperature fluctuations (Bidochka et al., 2001; Vega et al., 2012). Intense exposure to solar radiation can be detrimental to any organism (Solomon, 2008). Four hours of exposure to UV-B light can be enough to considerably reduce colony development and conidial viability, as well as delay conidial germination and cause conidial inactivation of *Metarhizium* spp. isolates (Braga et al., 2001a,b,c). Temperature is also an important factor affecting fungal propagules. Walstad et al. (1970) reported that the optimum temperature range for isolates of *M. anisopliae* s.l. was 25–30°C, but most isolates could germinate and sporulate between 15 and 35°C. Temperatures outside the latter range can decrease growth rates and reduce the virulence of *Metarhizium* isolates (Thomas and Jenkins, 1997; Ekesi et al., 1999; Inglis et al., 2001; Tumuhaise et al., 2018; Acheampong et al., 2020a).

Fungal propagules present above ground, such as in phylloplanes, are more exposed to the harmful effects of solar radiation and high temperatures than propagules in the soil environment. Therefore, it should be expected that natural selection will favor fungi that are more tolerant to these abiotic factors above ground as adaptive traits. Consequently, phylloplane-inhabiting fungal isolates are expected to exhibit higher survival rates than those inhabiting the soil after the same exposure time to UV light and high temperatures.

The adverse effects of these abiotic factors on entomopathogenic fungal isolates are considered significant obstacles to their application as biopesticides due to a reduced probability of establishing epizootics (Braga et al., 2001a). Moreover, tolerance to UV radiation or high temperatures may be responsible for the niche differentiation between the three *Metarhizium* species mentioned, i.e., *M. anisopliae* subclade Mani 2 acting mostly as an entomopathogen above ground, and *M. brunneum* and *M. robertsii* primarily associated with plant roots below ground.

Based on these assumptions, we selected isolates of the *M. anisopliae* subclade Mani 2, *M. robertsii*, and *M. brunneum* from different hosts and habitats in Brazil to evaluate their relative conidial survival after exposure to UV-B radiation, and conidial germination and mycelial growth rates under different temperature regimes. Information about these responses is useful for expanding the knowledge about the ecology of *Metarhizium* species and understanding how particular fungal isolates can survive under unfavorable abiotic conditions that can affect their abundance and distribution. It was expected that isolates of *M. anisopliae* subclade Mani 2 would exhibit the highest tolerance to UV-B radiation and elevated temperatures since the members of this subclade are most frequently found infecting insects above ground, thereby being more exposed to these abiotic factors. In contrast, *M. robertsii* and *M. brunneum* isolates, more often recovered from the soil environment and less exposed than *M. anisopliae*, would exhibit the lowest tolerance to UV-B and show the most activity at relatively low temperatures compared to *M. anisopliae*. Furthermore, isolates from lower latitudes of Brazil, where solar irradiation is stronger, and temperatures are usually high, should also be more tolerant to UV-B radiation and high temperatures than those from higher latitudes.

## Materials and Methods

### Fungal Isolates

Twelve isolates of *Metarhizium* spp. (six of *M. anisopliae* subclade Mani 2, three of *M. robertsii*, and three of *M. brunneum*) were selected from the Entomopathogen Collection “Prof. Sérgio Batista Alves,” of the Laboratory of Pathology and Microbial Control of Insects, at the Luiz de Queiroz College of Agriculture, University of São Paulo, Piracicaba, State of São Paulo, Brazil. Information about the collection site and origin of the isolates is shown in Table 1. Isolates ESALQ 1426 and ESALQ 1635 (*M. robertsii*) and all isolates of *M. anisopliae* were identified by Rezende et al. (2015). Isolate ESALQ 5168 (*M. robertsii*) and all isolates of *M. brunneum* were identified by Iwanicki (2015) and Iwanicki et al. (2019). Identification in all studies were based on sequencing of the 5′ EF-1α region.

**Table 1:** List of isolates used in the study, all deposited in the Entomopathogen Collection “Prof. Sérgio Batista Alves,” ESALQ/USP, Piracicaba, State of São Paulo, Brazil.

**Species**	**Isolate code**	**Origin**	**Collection site (City, State)**	**Latitude**
*Metarhizium anisopliae*	ESALQ 43	Hemiptera: Cercopidae	Flexeiras, Alagoas	9°16′ S
s.str. subclade Mani 2	ESALQ 1116	Coleoptera: Scarabaeidae	Piracicaba, São Paulo	22°43′ S
	ESALQ 1641	Hemiptera: Cercopidae	Boca da Mata, Alagoas	9°38′ S
	ESALQ 1076	Meadow soil	Arapongas, Paraná	23°25′ S
	ESALQ 1175	Meadow soil	Córrego Rico, São Paulo	21°15′ S
	ESALQ 1604	Biotech G, Biotech® Controle Biológico (commercial isolate)	Unknown	Unknown
*Metarhizium robertsii*	ESALQ 1426	Soybean soil	Londrina, Paraná	23°18′ S
	ESALQ 1635	Native forest soil	Delmiro Gouveia, Alagoas	9°23′ S
	ESALQ 5168	Coleoptera: Scarabaeidae	Iracemápolis, São Paulo	22°34′ S
*Metarhizium brunneum*	ESALQ 5022	Sugarcane soil	Iracemápolis, São Paulo	22°34′ S
	ESALQ 5286	Sugarcane soil	Iracemápolis, São Paulo	22°34′ S
	ESALQ 5181	Sugarcane root	Iracemápolis, São Paulo	22°34′ S

### Effect of UV-B Radiation Exposure

The isolates were grown in Petri dishes containing culture medium PDAY—Potato Dextrose Agar (Difco Laboratories, Sparks, MD, USA) enriched with yeast extract (2.5 g L^−1^) (KASVI, São José dos Pinhais, PR, Brazil)—and held in B.O.D. incubator (Biological Oxygen Demand) for 10 days (25 ± 1°C, 12 h photophase). After this period, conidia were harvested to prepare suspensions of each isolate (concentration: 10^6^ conidia mL^−1^) using sterile distilled water plus 0.05% Tween 80. Aliquots of 150 μL were inoculated covering the four central quadrants of Rodac Petri dishes (Replicate Organism Detection and Counting, 60 × 10 mm; J Prolab, São José dos Pinhais, PR, Brazil) containing 5 mL of PDAY plus 0.1% v/v Derosal 500 SC (Carbendazim, Bayer CropScience, SP, Brazil), a fungicide that has fungistatic properties at low concentrations. The plates were kept open in a laminar flow cabinet until all the liquid evaporated.

The experiment was conducted in a wooden box with four fluorescent lamps UVB-313EL (Q-Lab Corporation, USA), with peak irradiation corresponding to a wavelength of 313 nm (equivalent to UV-B light) and mean irradiation values of 659.54 mW m^−2^ or 2.38 kJ m^−2^. Before the exposure experiment, the lamps were turned on for 30 min to generate a stable irradiation level. The plates were then placed in the box and covered with an acetate sheet to prevent exposure to wavelengths below 290 nm, which includes UV-C (280 nm). The temperature inside the box was 27 ± 1°C during the experimental exposures. The experiment was performed using a randomized complete block design, in which all treatments were repeated three times at the same conditions. In each of the replicates (blocks), separate conidial suspensions of each isolate were prepared and used for inoculation in five plates of each isolate corresponding to each of the five exposure times (0, 2, 4, 6, and 8 h), totalizing 12 isolates × 5 exposure time (hours) × 3 blocks (replicates) = 180 observations. Plates representing the control were not exposed to UV-B (time = 0 h), while the other plates were exposed for 2, 4, 6, or 8 h (irradiation doses corresponding to 4.76, 9.52, 14.28, and 19.04 kJ m^−2^, respectively). Every 2 h, the respective plate of each isolate was transferred to an incubator (25 ± 1°C, 12 h photophase). The incubation time was 24 h for control plates (otherwise germination tubes grow and it becomes impossible to count germinated conidia) and 48 h for exposed plates to allow DNA repair and germination of conidia. Viabilities were then evaluated, counting germinated and non-germinated conidia under a light microscope and at least 200 conidia per plate; a propagule was considered germinated when the length of its germ tube was equal to or higher than its diameter.

### Effects of Temperature

#### Mycelial Growth

The isolates were cultivated in culture medium PDA (Potato Dextrose Agar; Difco Laboratories, Sparks, MD, USA) and held in B.O.D. incubator (25 ± 1°C, 12 h photophase) for 3 days. After this period, mycelial discs (ϕ = 1 cm) were made using a cork borer and transferred to the center of new Petri dishes (ϕ = 9 cm) containing PDA. There were five plates (replicates) per treatment, consisting of 12 isolates at each of the five temperature regimes (5 plates × 5 temperatures = 25 plates per isolate). The plates were sealed with parafilm and incubated in B.O.D., in the dark, in five temperature regimes: 15°C constant, 20°C constant, 25°C constant, 33°C constant, and 33°C for 8 h and 20°C for 16 h. A completely randomized design was used. Two orthogonal axes were drawn at the bottom of the plates to serve as a reference. The diameter of the colonies was measured daily for 12 days using a ruler. The bioassay was repeated three times.

#### Survival of Conidia

Eleven isolates were cultivated on PDAY and placed in B.O.D. incubator (25 ± 1°C, 12 h photophase), while the isolate ESALQ 1635 (*M. robertsii*), which exhibited poor sporulation on PDAY, was grown only on PDA. After 10 days, conidia were harvested with a spatula, dried in a desiccator with silica (relative humidity—RH <20%; water activity—a_w_ ≤ 0.3) and placed in Eppendorf tubes, which were vacuum packed to avoid interference of humidity; each tube contained 0.1 g of pure conidia. There were three Eppendorf tubes (replicates) per treatment, consisting of 12 isolates for each temperature regime (3 tubes × 3 temperatures = 9 tubes per isolate). A completely randomized design was used. The tubes were placed in B.O.D. incubators in the dark at 20, 25, or 40°C. After 12 days, the tubes were placed inside a laminar flow cabinet and kept open for 30 min to allow slow hydration of conidia and avoid imbibition damage. Later, viabilities were evaluated according to the protocol of Oliveira et al. (2015). Briefly, suspensions of each tube (concentration: 10^6^ conidia mL^−1^) were prepared, and aliquots of 150 μL were inoculated in Rodac Petri dishes containing PDA with an antibiotic (Pentabiótico: 500 mg L^−1^; composed of benzathine benzylpenicillin, procaine benzylpenicillin, benzylpenicillin potassium, dihydrostreptomycin base, and streptomycin base) and a fungistatic (0.1% v/v Derosal 500 SC), covering the four central quadrants; plates remained open in a laminar flow cabinet until all the liquid evaporated. The plates were then closed and incubated in B.O.D. (25 ± 1°C, 12 h photophase) for 24 h, after which viabilities were analyzed by counting germinated conidia.

### Statistical Analyses

For analyses of the three experiments' data, generalized linear models (GLMs) were used, allowing analysis of the normal and proportional responses, as long as the distribution is part of the exponential family (Nelder and Wedderburn, 1972).

All models were selected using the half-normal plot (Moral et al., 2017), and a likelihood-ratio test allowed us to compare similarities between isolates in different conditions (Demétrio et al., 2014; Fatoretto et al., 2018). In the UV-B experiment, the quasi-binomial model was proposed and allowed to capture any overdispersion present in the data (Demétrio et al., 2014). For the experiment of temperature affecting the survival of conidia, the theory of combined models was used, particularly the beta-binomial model (Molenberghs et al., 2017). These models can capture the data's overdispersion and allow the addition of the random effects that model the correlation within individuals. The model was adjusted using the GAMLSS package (Rigby and Stasinopoulos, 2005). Modeling of mycelial growth data was performed considering normal data, the dependence of the parcels (plates) inside the same B.O.D. incubator, and between parcels, which were the measurements over time of the same plate. A model was designed for each temperature, considering measurements from the 4th day. All analyses were performed using the software R (R Core Team, 2020).

## Results

### Effect of UV-B on Conidial Germination of Isolates

**Figure 1:**
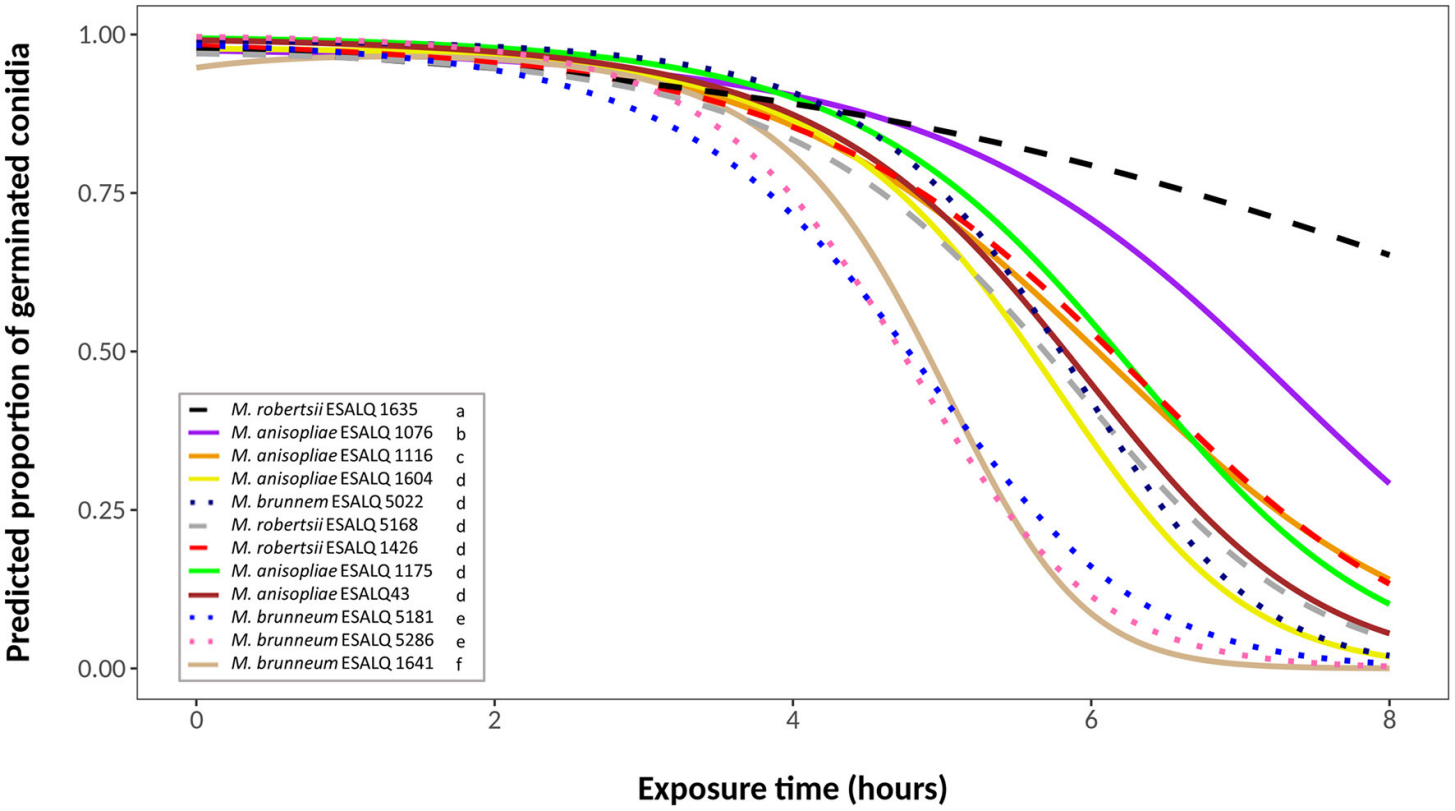
Predicted proportion of germinated conidia of isolates of *Metarhizium robertsii, M. brunneum*, and *M. anisopliae* s.str. Mani 2 after UV-B exposure for 2, 4, 6 or 8 h, adopting a quasi-binomial model with logit link function (α = 0.05%).

Due to considerable variability in the data, the binomial model did not present a goodness-of-fit. The best fitted model was a quasi-binomial model with a quadratic linear predictor for exposure time. In this model, conidial viability is described by separate curves, in which each fungus has distinct initial proportions (different intercepts) and different slopes (decay form) for each time (Figure 1).

Germination of control plates (no UV-B exposure) ranged between 97 and 100%. UV-B light had little effect on conidia germination after 2 h of exposure, and germination rates were still above 70% for all isolates after 4 h. After 6 h of exposure, most of the isolates started to show a substantial decline in conidial germination. By the end of the experimental time (8 h of exposure), nine isolates exhibited germination below 5% (Figure 1). Isolates *M. robertsii* ESALQ 1426, *M. brunneum* ESALQ 5181 and ESALQ 5286, and *M. anisopliae* ESALQ 1175 and ESALQ 1641 reached complete inactivation of conidia at 8 h, while isolate *M. robertsii* ESALQ 1635 was the most tolerant to UV-B radiation, being the only isolate to retain more than 50% of germination after 8 h.

Since some isolates exhibited similar curves, likelihood-ratio tests were conducted to identify possible similarities between their viability. Six groups were defined (α = 0.05), in order of UV-B tolerance (highest to lowest): (i) *M. robertsii* ESALQ 1635; (ii) *M. anisopliae* ESALQ 1076; (iii) *M. anisopliae* ESALQ 1116; (iv) *M. anisopliae* isolates ESALQ 43, ESALQ 1175, and ESALQ 1604, *M. robertsii* isolates ESALQ 1426 and ESALQ 5168, and *M. brunneum* isolate ESALQ 5022; (v) *M. brunneum* isolates ESALQ 5181 and ESALQ 5286; and (vi) *M. anisopliae* ESALQ 1641 (Figure 1).

### Effect of Temperature on Mycelial Growth of Isolates

**Figure 2:**
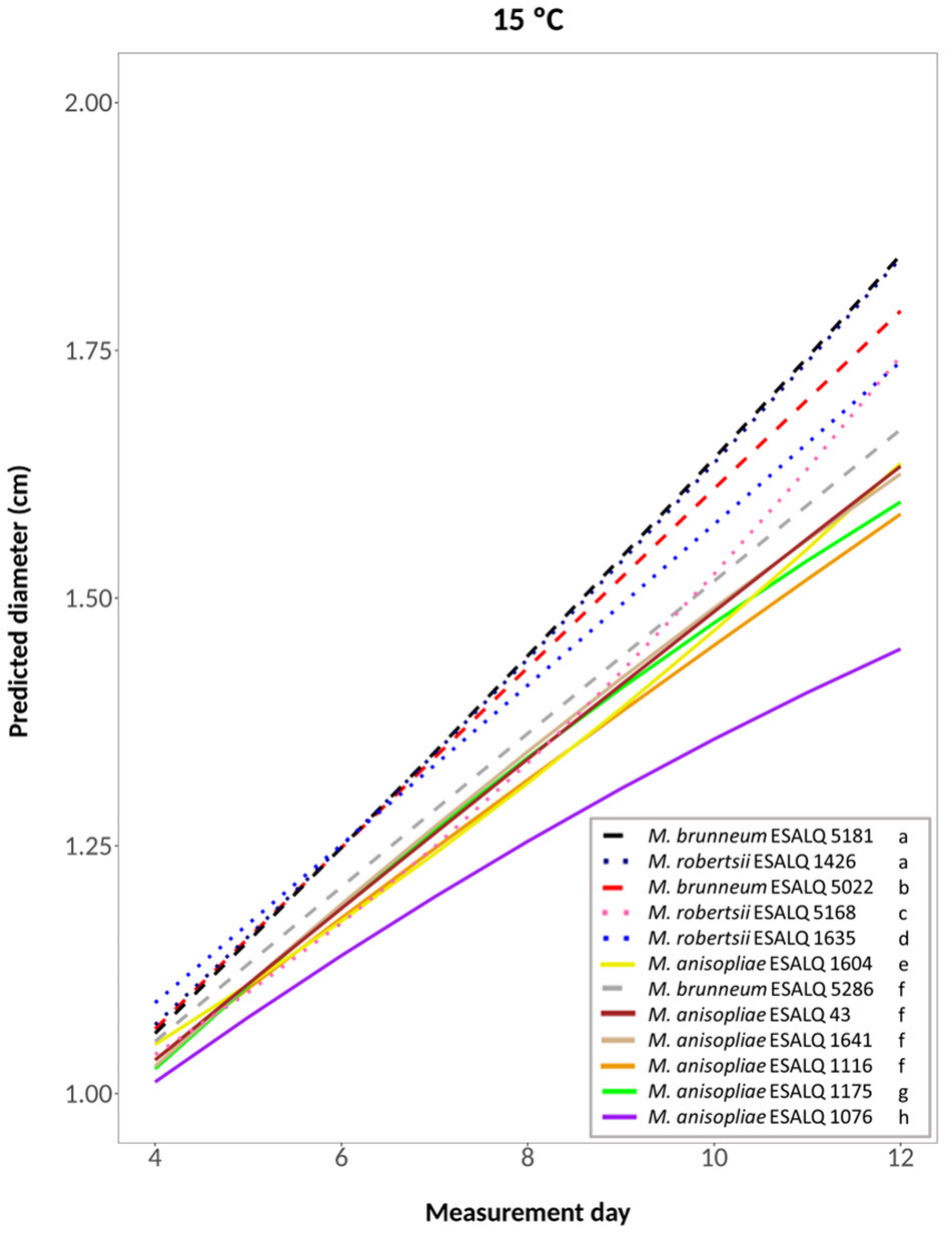
Predicted diameter of colonies of isolates of *Metarhizium robertsii, M. brunneum*, and *M. anisopliae* s.str. Mani 2 after 12 days at 15°C constant, adopting a quadratic model (α = 0.05%).

The selected model for the analysis of mycelial growth of the 12 isolates at 15°C was a quadratic regression model; the hypothesis that it well-represents the data was accepted after performing the lack of fit test, considering the isolate as a factor and measurement day as a quadratic term (*P* = 0.4058; Figure 2). Through a likelihood-ratio test to compare the slope (growth rate) and the intercept between curves, isolates were grouped according to their growth rate and/or intercept. The highest growth rates were achieved by isolates *M. brunneum* ESALQ 5181 and *M. robertsii* ESALQ 1426 (Group 1), followed by *M. brunneum* ESALQ 5022 (Group 2), *M. robertsii* ESALQ 5168 (Group 3), and ESALQ 1635 (Group 4). The six *M. anisopliae* isolates had the lowest growth rates at this temperature, although, comparing the curves, *M. brunneum* ESALQ 5286 was grouped with three of them (ESALQ 43, ESALQ 1641, and ESALQ 1116).

**Figure 3:**
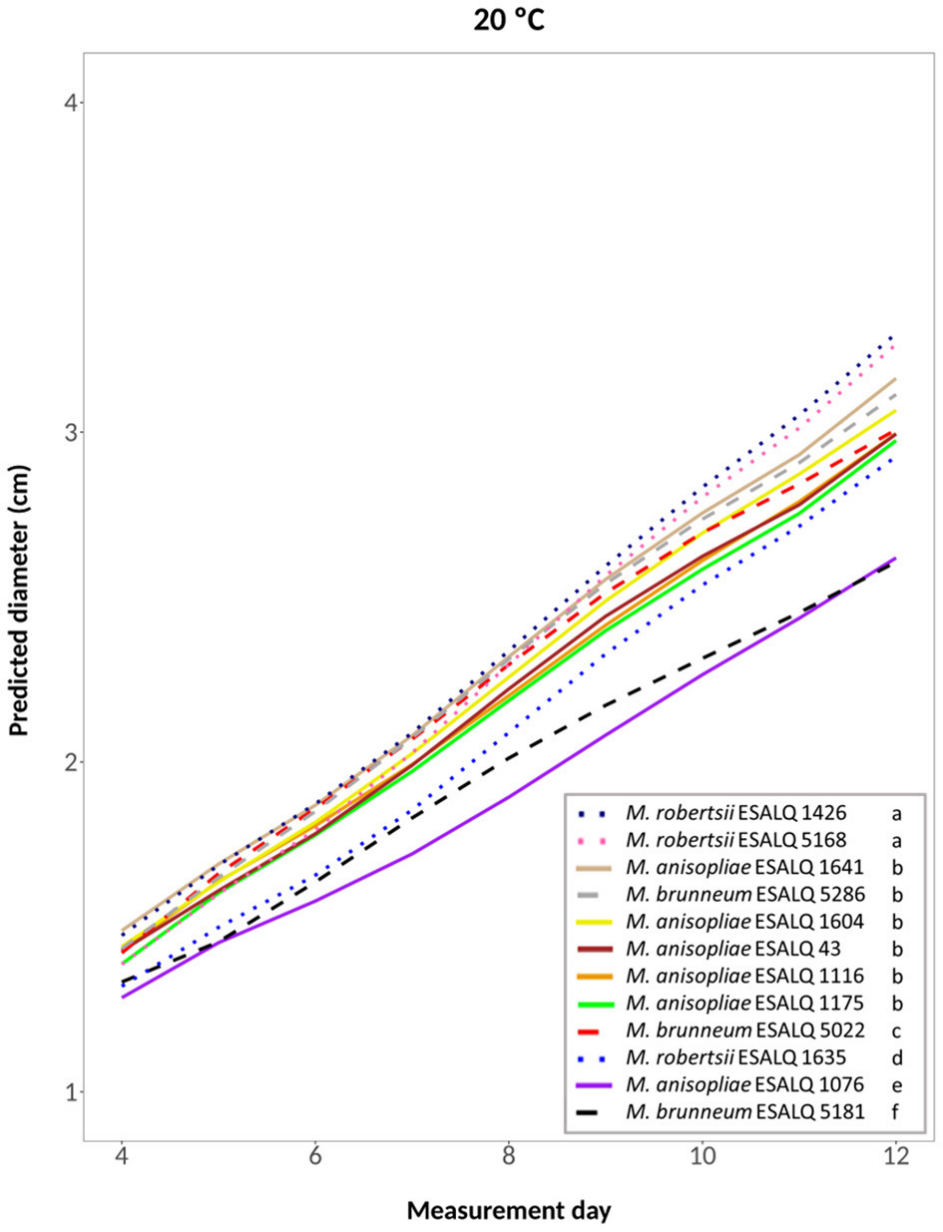
Predicted diameter of colonies of isolates of *Metarhizium robertsii, M. brunneum*, and *M. anisopliae* s.str. Mani 2 after 12 days at 20°C constant, adopting a fifth-degree polynomial model (α = 0.05%).

A fifth-degree polynomial model was selected for the temperature 20°C constant (hypothesis accepted against a whole model: *P* = 0.8095; Figure 3). At this temperature, *M. robertsii* ESALQ 1426 and ESALQ 5168 had the highest growth rates, being grouped. Six other isolates were also grouped: *M. brunneum* ESALQ 5286 and *M. anisopliae* ESALQ 1641, ESALQ 1604, ESALQ 43, ESALQ 1175, and ESALQ 1116. The other isolates were considered individual groups. Growth was lowest for *M. anisopliae* ESALQ 1076 and *M. brunneum* ESALQ 5181.

**Figure 4:**
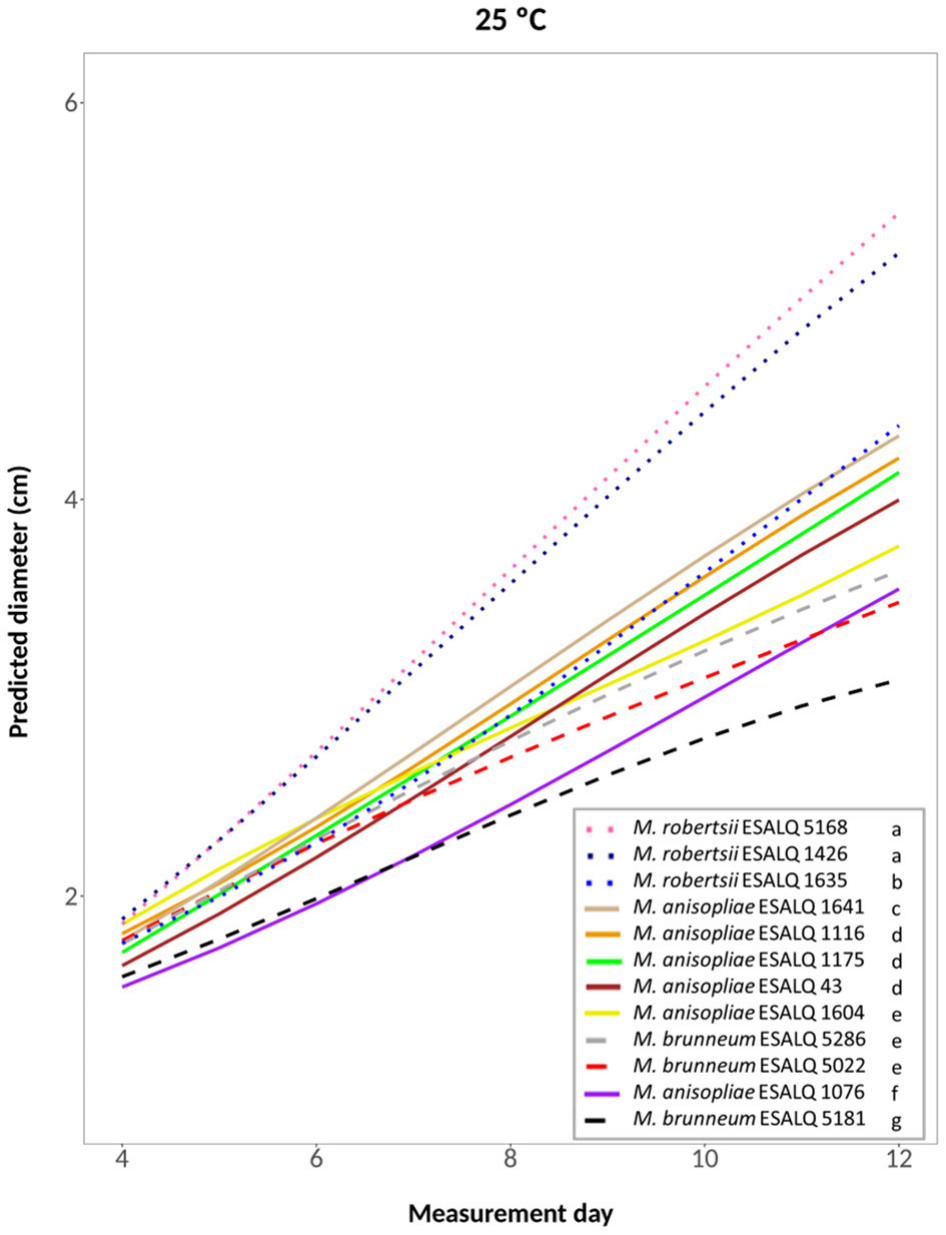
Predicted diameter of colonies of isolates of *Metarhizium robertsii, M. brunneum*, and *M. anisopliae* s.str. Mani 2 after 12 days at 25°C constant, adopting a cubic model (α = 0.05%).

**Figure 5:**
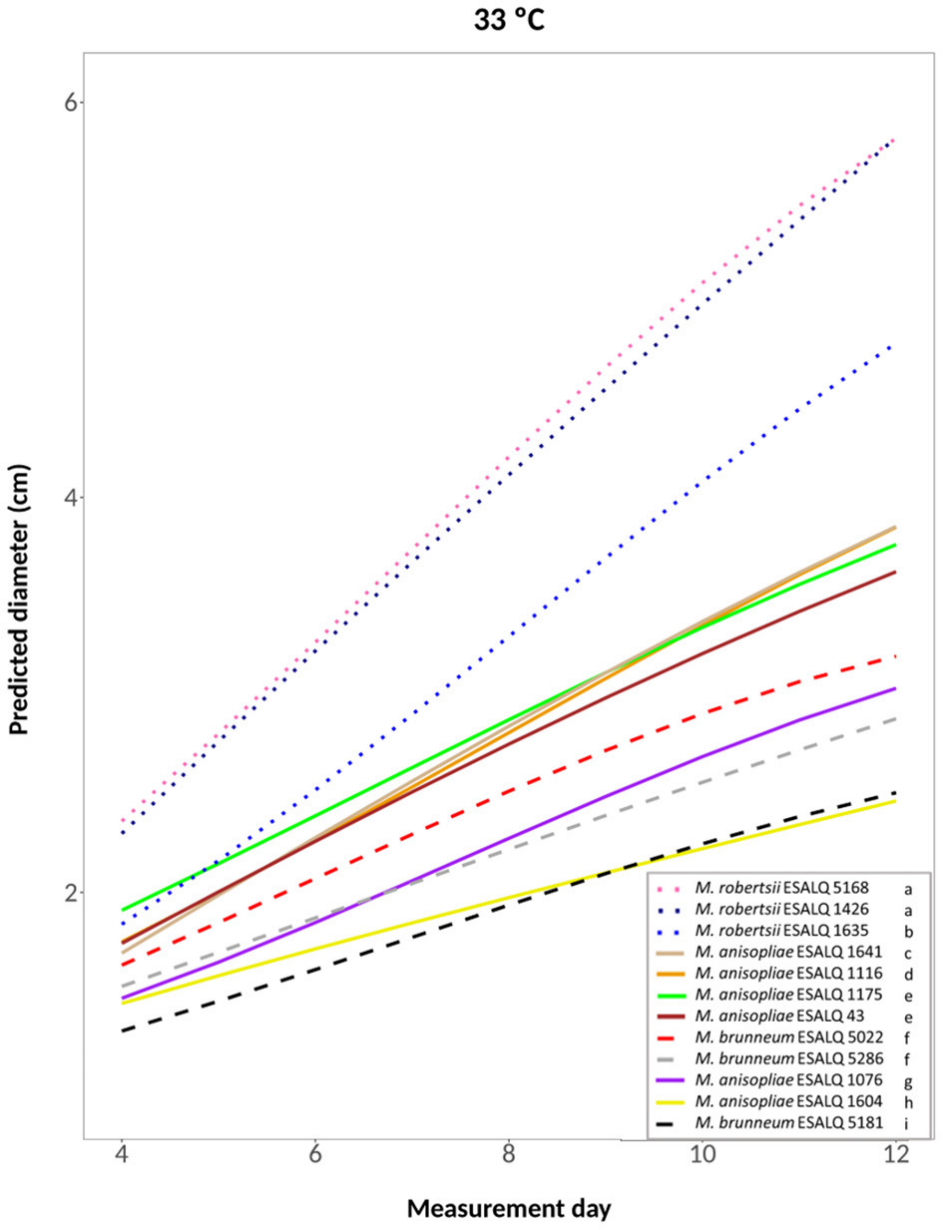
Predicted diameter of colonies of isolates of *Metarhizium robertsii, M. brunneum*, and *M. anisopliae* s.str. Mani 2 after 12 days at 33°C constant, adopting a cubic model (α = 0.05%).

A cubic model was selected for the treatments at 25°C constant, at 33°C constant and the one alternating 20 and 33°C (hypotheses accepted against a whole model, respectively: *P* = 0.2309, Figure 4; *P* = 0.3961, Figure 5; *P* = 0.3904, Supplementary Figure 1). For these treatments, the three *M. robertsii* isolates had the fastest growth, with ESALQ 1426 and ESALQ 5168 being grouped with the highest rates under these three temperature conditions. Similarly, the three temperature regimes resulted in five isolates (*M. brunneum* ESALQ 5022, ESALQ 5181 and ESALQ 5286, and *M. anisopliae* ESALQ 1076 and ESALQ 1604) consistently showing the lowest growth rates (Figures 4–5, Supplementary Figure 1).

In summary, the three *M. robertsii* isolates achieved higher growth at 33°C, while isolates of *M. brunneum* and *M. anisopliae* had optimal growth rates at 25°C. For all 12 isolates, mycelial growth was slower at 15°C. A comparison of growth rates for each isolate is shown in Supplementary Figure 2.

### Temperature Affecting the Survival of Conidia

The beta-binomial model, with the parameters of the distribution location (mean) and scale (variance), brought a plausible realization of the data of temperature effects on conidial survival (Table 2). All 12 isolates maintained high viabilities at 20 and 25°C, but the same did not occur at 40°C: conidia of all *M. brunneum* and *M. robertsii* isolates exhibited very low proportions of survival, while the conidia of the *M. anisopliae* isolates showed higher tolerance.

**Table 2:** Survival of conidia (percentage) of *Metarhizium robertsii* (*Mr*), *M. brunneum* (*Mb*), and *M. anisopliae* s.str.

**Conidial survival at 20** ^**°**^ **C**				**Conidial survival at 25** ^**°**^ **C**				**Conidial survival at 40** ^**°**^ **C**			
**Species/Isolate**		**%**		**Species/Isolate**		**%**		**Species/Isolate**		**%**	
*Mb*	ESALQ 5286	94.5	a	*Ma*	ESALQ 43	94.3	a	*Ma*	ESALQ 1175	66.6	a
*Ma*	ESALQ 43	94.1	a	*Mb*	ESALQ 5286	93.7	a	*Ma*	ESALQ 1641	61.1	a
*Ma*	ESALQ 1604	93.5	a	*Mb*	ESALQ 5022	92.7	a	*Ma*	ESALQ 1076	44.1	b
*Mb*	ESALQ 5181	93.4	a	*Mb*	ESALQ 5181	90.7	a	*Ma*	ESALQ 43	34.4	b
*Ma*	ESALQ 1076	91.7	a	*Ma*	ESALQ 1175	89.6	a	*Ma*	ESALQ 1116	26.6	b
*Mr*	ESALQ 1426	91.3	a	*Ma*	ESALQ 1116	89.5	a	*Ma*	ESALQ 1604	8.9	c
*Ma*	ESALQ 1641	91.3	a	*Mr*	ESALQ 1426	89.2	a	*Mr*	ESALQ 1635	5.1	c
*Mb*	ESALQ 5022	90.8	a	*Ma*	ESALQ 1604	89.1	a	*Mb*	ESALQ 5286	4.7	c
*Ma*	ESALQ 1175	90.6	a	*Mr*	ESALQ 1635	89.0	a	*Mb*	ESALQ 5022	3.8	c
*Mr*	ESALQ 1635	90.4	a	*Ma*	ESALQ 1076	89.0	a	*Mr*	ESALQ 5168	1.8	d
*Ma*	ESALQ 1116	89.8	a	*Mr*	ESALQ 5168	86.9	a	*Mr*	ESALQ 1426	1.7	d
*Mr*	ESALQ 5168	84.5	b	*Ma*	ESALQ 1641	86.4	a	*Mb*	ESALQ 5181	0.5	d

*(Ma) after 12 days of incubation at 20°, 25°, or 40°C, adopting a beta-binomial distribution (α = 0.05%). Different letters in the same column indicate significant differences between isolates for each temperature*.

A likelihood-ratio test was conducted for each temperature to check for similarities between isolates and according to the experimental design. The isolates were separated into two groups for the treatments at 20°C (hypothesis accepted, *P* = 0.4628): one composed of the isolate *M. robertsii* ESALQ 5168, with a slightly lower predicted proportion of germination than the other 11 isolates. At 25°C, no differences were found among the 12 isolates (hypothesis accepted, *P* = 0.6220). At 40°C, it was possible to separate the isolates into four groups (hypothesis accepted, *P* = 0.0652) (in order of high to low tolerance): (i) *M. anisopliae* ESALQ 1175 and ESALQ 1641; (ii) *M. anisopliae* ESALQ 1076, ESALQ 43, and ESALQ 1116; (iii) *M. anisopliae* ESALQ 1604, *M. robertsii* ESALQ 1635, *M. brunneum* ESALQ 5286, and ESALQ 5022; and (iv) *M. robertsii* ESALQ 5168 and ESALQ 1426, *M. brunneum* ESALQ 5181.

In order to give an overview of all results, Table 3 contains a summary of the performance of each of the 12 isolates across all the parameters tested and shows that isolates of the same species can exhibit very different responses depending on environmental variables.

**Table 3:** Performance of each isolate of *Metarhizium robertsii, M. brunneum*, and *M. anisopliae* s.str. in three experiments involving exposure to UV-B radiation for up to 8 h, mycelial growth after 12 days under different temperature regimes (15°C constant, 20°C constant, 25°C constant, 33°C constant, and 20°C for 16 h + 33°C for 8 h), and conidial survival after 12 days at three temperatures (20°, 25°, and 40°C).

**UV-B tolerance**	**Mycelial Growth**	**Mycelial Growth**	**Mycelial Growth**	**Mycelial Growth**	**Mycelial Growth**	**Conidial Survival**	**Conidial Survival**	**Conidial Survival**
	**15^**°**^C constant**	**20^**°**^C constant**	**25^**°**^C constant**	**33^**°**^C constant**	**20^**°**^C/16 h + 33^**°**^C/8 h**	**20^**°**^C**	**25^**°**^C**	**40^**°**^C**
ESALQ 1635	ESALQ 5181	ESALQ 1426	ESALQ 5168	ESALQ 5168	ESALQ 5168	ESALQ 5286	ESALQ 43	ESALQ 1175
ESALQ 1076	ESALQ 1426	ESALQ 5168	ESALQ 1426	ESALQ 1426	ESALQ 1426	ESALQ 43	ESALQ 5286	ESALQ 1641
ESALQ 1116	ESALQ 5022	ESALQ 1641	ESALQ 1635	ESALQ 1635	ESALQ 1635	ESALQ 1604	ESALQ 5022	ESALQ 1076
ESALQ 1604	ESALQ 5168	ESALQ 5286	ESALQ 1641	ESALQ 1641	ESALQ 1175	ESALQ 5181	ESALQ 5181	ESALQ 43
ESALQ 5022	ESALQ 1635	ESALQ 1604	ESALQ 1116	ESALQ 1116	ESALQ 1641	ESALQ 1076	ESALQ 1175	ESALQ 1116
ESALQ 5168	ESALQ 1604	ESALQ 43	ESALQ 1175	ESALQ 1175	ESALQ 1116	ESALQ 1426	ESALQ 1116	ESALQ 1604
ESALQ 1426	ESALQ 5286	ESALQ 1116	ESALQ 43	ESALQ 43	ESALQ 43	ESALQ 1641	ESALQ 1426	ESALQ 1635
ESALQ 1175	ESALQ 43	ESALQ 1175	ESALQ 1604	ESALQ 5022	ESALQ 5022	ESALQ 5022	ESALQ 1604	ESALQ 5286
ESALQ 43	ESALQ 1641	ESALQ 5022	ESALQ 5286	ESALQ 5286	ESALQ 5286	ESALQ 1175	ESALQ 1635	ESALQ 5022
ESALQ 5181	ESALQ 1116	ESALQ 1635	ESALQ 5022	ESALQ 1076	ESALQ 1076	ESALQ 1635	ESALQ 1076	ESALQ 5168
ESALQ 5286	ESALQ 1175	ESALQ 1076	ESALQ 1076	ESALQ 1604	ESALQ 1604	ESALQ 1116	ESALQ 5168	ESALQ 1426
ESALQ 1641	ESALQ 1076	ESALQ 5181	ESALQ 5181	ESALQ 5181	ESALQ 5181	ESALQ 5168	ESALQ 1641	ESALQ 5181

*In each column, groupings are delimited by closed cells (α = 0.05%). Tolerance order decreases from the top to the bottom of the table. Isolates of M. robertsii are colored in gray, M. brunneum in blue and M. anisopliae in green*.

## Discussion

Solar radiation, especially of the UV-B type, is very harmful to fungal propagules, significantly affecting their survival and efficacy against insects in the environment (Ignoffo and Garcia, 1992; Inglis et al., 2001; Fernandes et al., 2015; Acheampong et al., 2020b). Previous reports (e.g., Braga et al., 2001a,c; Fernández-Bravo et al., 2017) showed that 2 h of exposure to UV-B light (irradiances of 920 or 1,200 mW m^−2^, corresponding to total doses of 6.6 or 8.6 kJ m^−2^, respectively) was enough to substantially reduce conidial culturability (in some cases, more than 50%) of *Metarhizium* spp. isolates, including some of Brazilian origin. In the present study, it was only after 6 h of exposure (total dose of 14.28 kJ m^−2^) that the germination rates were reduced for almost all 12 isolates of *Metarhizium* spp. from Brazil, with five of them becoming completely inactivated after 8 h of exposure (total dose of 19.04 kJ m^−2^).

Considerable intra- and inter-specific variability in UV-B tolerance was found among the 12 isolates. Similar observations were reported by Fargues et al. (1996), who compared the survival of conidia of isolates of *Metarhizium flavoviride, Beauveria bassiana, M. anisopliae* s.l., and *Cordyceps fumosorosea* (= *Isaria fumosorosea, Paecilomyces fumosoroseus*) and found different degrees of tolerance between and within species (e.g., for *B. bassiana* isolates, survival ranged from 0 to 100%). Furthermore, Fernandes et al. (2007) found a large range of resilience to UV-B radiation testing 59 *Beauveria* spp. isolates, while Huang and Feng (2009) reported variable tolerances among 20 *B. bassiana* isolates, based on UV-B lethal doses (LD_50_, LD_75_, and LD_95_; J cm^−2^) after irradiation.

When investigating the association between the latitude of origin of *Beauveria* spp. isolates (most of them from Brazil) and their UV tolerance, Fernandes et al. (2007) reported a significant inverse correlation, i.e., the most UV tolerant isolates were from regions of lower latitude, where solar irradiation is more intense than at higher latitudes. Braga et al. (2001c), working with isolates of *M. anisopliae* s.l., reported a similar correlation and stated that natural selection for UV-B tolerance must have occurred with these isolates. In the present study, no apparent latitudinal gradient in UV-B tolerance could be seen, as the isolate most susceptible to UV-B radiation (ESALQ 1641) was obtained in a region of lower latitude, while the second (ESALQ 1076) and third (ESALQ 1116) most tolerant isolates were collected in areas of higher latitudes. Likewise, Fargues et al. (1996) did not find a correlation between the geographic origin of *B. bassiana* isolates and UV-B tolerance.

Our results also indicate no apparent relationship between UV-B tolerance and the substrate of isolation or fungal species. Isolates obtained from different substrates (i.e., insect, soil or root) or of varying *Metarhizium* species were grouped by the similarity of their survival curves (Figure 1), while others originating from the same type of substrate had different angles, even if they were of the same species (e.g., *M. anisopliae* ESALQ 1076 and ESALQ 1175, both from meadow soil). This finding is in accordance with reports that used *Metarhizium* spp. and *Beauveria* spp., and a positive correlation involving UV-B tolerance and isolation substrate or fungal species has yet to be found (Fernandes et al., 2007; Fernández-Bravo et al., 2016, 2017).

One *M. robertsii* isolate (ESALQ 1635) was by far the most UV-B tolerant, and some isolates of the three species had similar conidial survival curves. This indicates that tolerance to UV-B radiation was not selected at the species level among those considered adapted for the above-ground environment. Evidence suggests that a difference in habitat (e.g., phylloplane vs. soil) does not influence the ability of isolates to resist elevated levels of UV radiation (Fernández-Bravo et al., 2016, 2017).

Many reports about the effects of temperature on *Metarhizium* spp. isolates state that the optimal temperature for growth ranges between 25 and 30°C (Ekesi et al., 1999; Dimbi et al., 2004; Zimmermann, 2007; Acheampong et al., 2020a). Our data for *M. anisopliae* and *M. brunneum* isolates corroborate this and show that the best temperature for their mycelial growth *in vitro* was 25°C. At temperatures higher than 30°C, isolates can grow, but there is a decrease in growth rate, and generally, growth ceases at 35°C (Ouedraogo et al., 1997; Acheampong et al., 2020a). These authors reported an isolate of *M. anisopliae* s.l. with an optimum growth temperature of 28–32°C. Welling et al. (1994) studied an isolate of *M. flavoviride* that achieved the highest growth at 30–34°C. Similarly, our three *M. robertsii* isolates had the highest growth rates at 33°C, indicating higher thermotolerance of the mycelium than for the *M. anisopliae* and *M. brunneum* isolates.

Overall, the three isolates of *M. robertsii* showed relatively high growth rates, the three isolates of *M. brunneum* had the lowest growth rates, and the six *M. anisopliae* isolates were intermediate. However, considerable intraspecific variability was found for the isolates of all three species. Ouedraogo et al. (1997) also reported within-species variability between isolates of *M. anisopliae* s.l. and *M. flavoviride*.

An association between thermotolerance and habitat of origin was reported for species such as *M. anisopliae* s.l., *M. flavoviride*, and *M. rileyi* (= *Nomuraea rileyi*), with isolates from tropical or subtropical regions being more tolerant and having higher growth rates at higher temperatures than isolates from temperate areas (Fargues et al., 1992; Vidal et al., 1997). The performance of the three *M. brunneum* isolates under different temperatures in this study, the high prevalence of this species in temperate zones such as North America and Europe (where temperatures are cooler), and their low occurrence in South America (Steinwender et al., 2014; Rezende et al., 2015) might indicate that this species is not adapted to tolerate high temperatures. Our isolates were obtained from tropical and subtropical regions in Brazil, and an association between thermotolerance and latitude of origin could not be found in our study (e.g., *M. robertsii* ESALQ 1426, from south Brazil, was more tolerant to 33°C than isolates from northeast Brazil). Other studies also reported the absence of a relationship between thermotolerance and isolate geographic origin (*Metarhizium* spp. and *B. bassiana*) (De Croos and Bidochka, 1999; Devi et al., 2005; Rangel et al., 2005). Furthermore, no association was found between tolerance and substrate of isolation (e.g., *M. robertsii* ESALQ 1426, from soil, and *M. robertsii* ESALQ 5168, from insects, had the highest growth rates and grouped at all temperatures except at 15°C).

Exposure time to certain temperatures is essential when considering the tolerance of an isolate. Welling et al. (1994) reported that two out of three *Metarhizium* spp. isolates showed higher growth in a temperature cycle of 16 h/25°C + 8 h/34°C compared to 34°C constant, and all of them were able to grow at temperatures above 40°C when the temperature cycle included a period of 16 h at 25°C, although with a decrease in growth rate compared to treatments with lower maximal temperatures (e.g., 25°C constant or cycle of 16 h/25°C + 8 h/30°C). In our study, we used a cycle of 8 h at 33°C and 16 h at 20°C, and even though the three isolates of *M. brunneum* and two of the *M. anisopliae* isolates did not grow well at 33°C constant, growth was numerically higher than with alternating temperatures.

The viabilities of dried conidia were not significantly affected by treatments at 20 or 25°C, varying in germination rates between 84 and 95% after 12 days, with only a slight reduction. On the other hand, there was an apparent effect of high temperature, as dried conidia of all 12 isolates showed a significant decrease in survival at 40°C. This lower viability can be explained by the fact that high temperatures can retard and inhibit conidial germination and cause growth termination (Walstad et al., 1970; Inglis et al., 2001). Conidia of two isolates of *M. anisopliae* s.l. submitted to a range of temperatures (−20 to 37°C) survived for longer periods in the lower range (Daoust and Roberts, 1983), and dried conidia of an isolate of *M. acridum* (reported as *M. flavoviride* by the authors) also had a higher germination percentage after being stored in colder conditions compared to storage at higher temperatures (Moore et al., 1996). Clerk and Madelin (1965) evaluated the conidial survival of an isolate of *M. anisopliae* s.l. at 8, 18, and 25°C under different RH conditions, and treatments at 25°C affected conidia the most. A reduction in the temperature of storage can increase the survival of conidia (Clerk and Madelin, 1965; Walstad et al., 1970).

In agreement with other studies (Fargues et al., 1997; Rangel et al., 2005), and similar to what we found about UV-B radiation, there was no apparent correlation observed between conidial thermotolerance and latitude or substrate of isolation. Our hypothesis relating this tolerance to fungal species was not fully met since ESALQ 1604 (*M. anisopliae*) exhibited similar conidial survival to ESALQ 1635 (*M. robertsii*), and ESALQ 5022 and ESALQ 5181 (*M. brunneum*). However, it is worth noting that the other five *M. anisopliae* isolates had significantly higher conidial survival proportions at 40°C. Rangel et al. (2005) found some variability in wet and dry heat tolerance of conidia among isolates of *Metarhizium* species exposed to 40 and 45°C, with *M. acridum* isolates exhibiting a significantly higher tolerance compared to isolates of *M. anisopliae* s.l., *M. robertsii*, or *M. brunneum*.

Although our experimental time was relatively short, it was enough to reduce conidial viabilities of some isolates below 5%, evidencing their susceptibility to high temperatures, an undesirable characteristic for biological control purposes in field conditions, especially in tropical countries (such as Brazil) where the air temperatures can reach above 40°C in the summer. Conidial survival at such high temperatures can be increased by drying and developing formulations with oils (Moore et al., 1996; Paixão et al., 2017).

Mycelia comprises a mass of hyphae related to the absorption of nutrients, vegetative growth, and plant associations. In contrast, conidia are asexual, infective spores capable of persisting in the environment in the absence of a host, forming reservoirs of inoculum (Evans and Hywel-Jones, 1997; Hesketh et al., 2010; Behie et al., 2012; Vega et al., 2012). Because of this, conidia are more subjected to adverse environmental conditions than mycelia; thus, they should exhibit increased tolerance to abiotic factors to improve their survival. Our data showed that mycelial growth rates of the three isolates of *M. robertsii* were higher at the highest temperature tested, but their conidia did not tolerate high temperature or UV-B radiation. On the other hand, the opposite result was obtained for most isolates of *M. anisopliae*, which produced conidia that showed increased tolerance to the stressful conditions tested, but the same could not be concluded for their mycelial growth *in vitro*. The fact that mycelia of *M. robertsii* isolates had optimum growth at 33°C, while their conidia were not tolerant to storage at 40°C (all three isolates) or UV-B radiation (ESALQ 1426 and ESALQ 5168), indicates a stronger selection pressure for mycelial growth rather than survival of conidia. This trait may have an essential role in their association with plant roots, where the active hyphae are the structure forming the association in the rhizosphere (Sasan and Bidochka, 2012; Behie and Bidochka, 2014). In contrast, conidia of *M. anisopliae* isolates could better tolerate the adverse experimental conditions, suggesting that adaptation for unfavorable conditions of these propagules is selected for, as they are important for survival in the environment and for infecting new hosts.

Although we found some variation in tolerance to UV-B radiation and high temperatures between isolates of the same species, similarities could also be seen, e.g., the three isolates of *M. robertsii* growing faster at 33°C or conidia of isolates of *M. anisopliae* better tolerating incubation at 40°C. The present study shows that isolates of each species represent biological plasticity and that isolates of the same species can exhibit very different responses to environmental variables, thus not making it possible to generalize results to the species level. A similar conclusion was reported by Canassa et al. (2020) concerning the ability of *Metarhizium* spp. isolates to interact with plants. Our study provides new insights into the ecology of entomopathogenic fungi and their adaptations for variation of important environmental abiotic factors that can impact the fungi abundance and distribution patterns. Knowledge of such adaptations can provide the foundation for future selection of candidate isolates with high environmental resilience for the development of biological control agents.

## Data Availability Statement

The datasets presented in this study can be found in online repositories. The names of the repository/repositories and accession number(s) can be found below: Repository Open Science Framework (OSF) doi: 10.17605/OSF.IO/EJXY3.

## Author Contributions

JC, NM, and íD: conceptualization. JC, NM, and íD: methodology. MF and CD: formal analysis. JC: investigation. JC: data curation. JC: writing—original draft preparation. JC, MF, CD, NM, and íD: writing—review and editing. NM and íD: supervision. All authors have read and agreed to the published version of the manuscript.

## Conflict of Interest

The authors declare that the research was conducted in the absence of any commercial or financial relationships that could be construed as a potential conflict of interest.
