# Supplementary material for: UV-B Radiation Tolerance and Temperature-Dependent Activity Within the Entomopathogenic Fungal Genus Metarhizium in Brazil
Source: Front Fungal Biol. 2021 Mar 8;2:645737. doi: 10.3389/ffunb.2021.645737 (PMC10512313; doi:10.3389/ffunb.2021.645737)
Supplement: Supplementary file 1 [file Data_Sheet_1.ZIP › Supplementary material.docx]

**Figure S1.** Predicted diameter of colonies of isolates of *Metarhizium robertsii*, *M. brunneum* and *M. anisopliae s.s.* Mani 2 after 12 days in a cycle of 20 °C for 16 h and 33 °C for 8 h, adopting a cubic model (α = 0.05%).

**Figure S2.** Comparison of the predicted diameters of colonies of isolates of *Metarhizium robertsii* (*Mr*), *M. brunneum* (*Mb*), and *M. anisopliae s.s.* (*Ma*) after 12 days in 5 different temperature conditions (15 °C constant, 20 °C constant, 25 °C constant, 33 °C constant, cycle of 20 °C for 16 h and 33 °C for 8 h).
