# Supplementary figures and images for: UV-B Radiation Tolerance and Temperature-Dependent Activity Within the Entomopathogenic Fungal Genus Metarhizium in Brazil
Source: Front Fungal Biol. 2021 Mar 8;2:645737. doi: 10.3389/ffunb.2021.645737 (PMC10512313; doi:10.3389/ffunb.2021.645737)

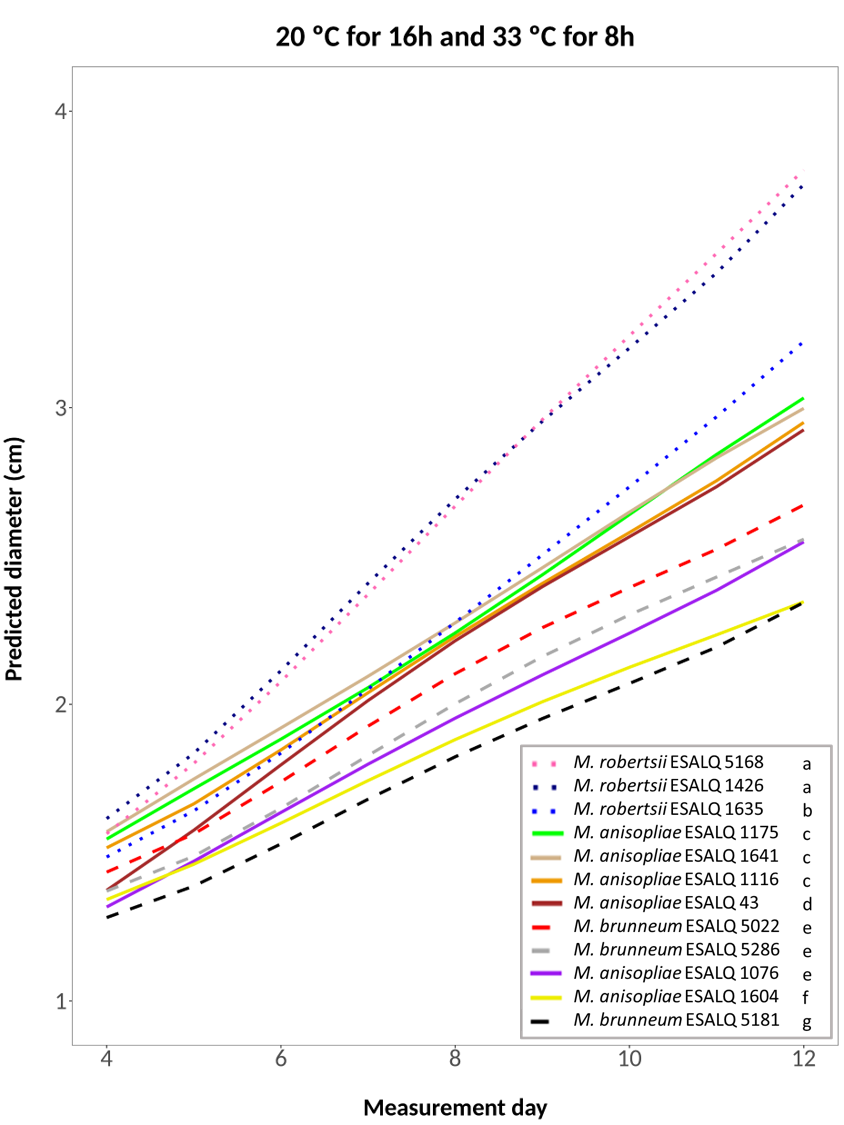

Supplement: Supplementary file 1 [file Data_Sheet_1.ZIP › Figure S1.tif]

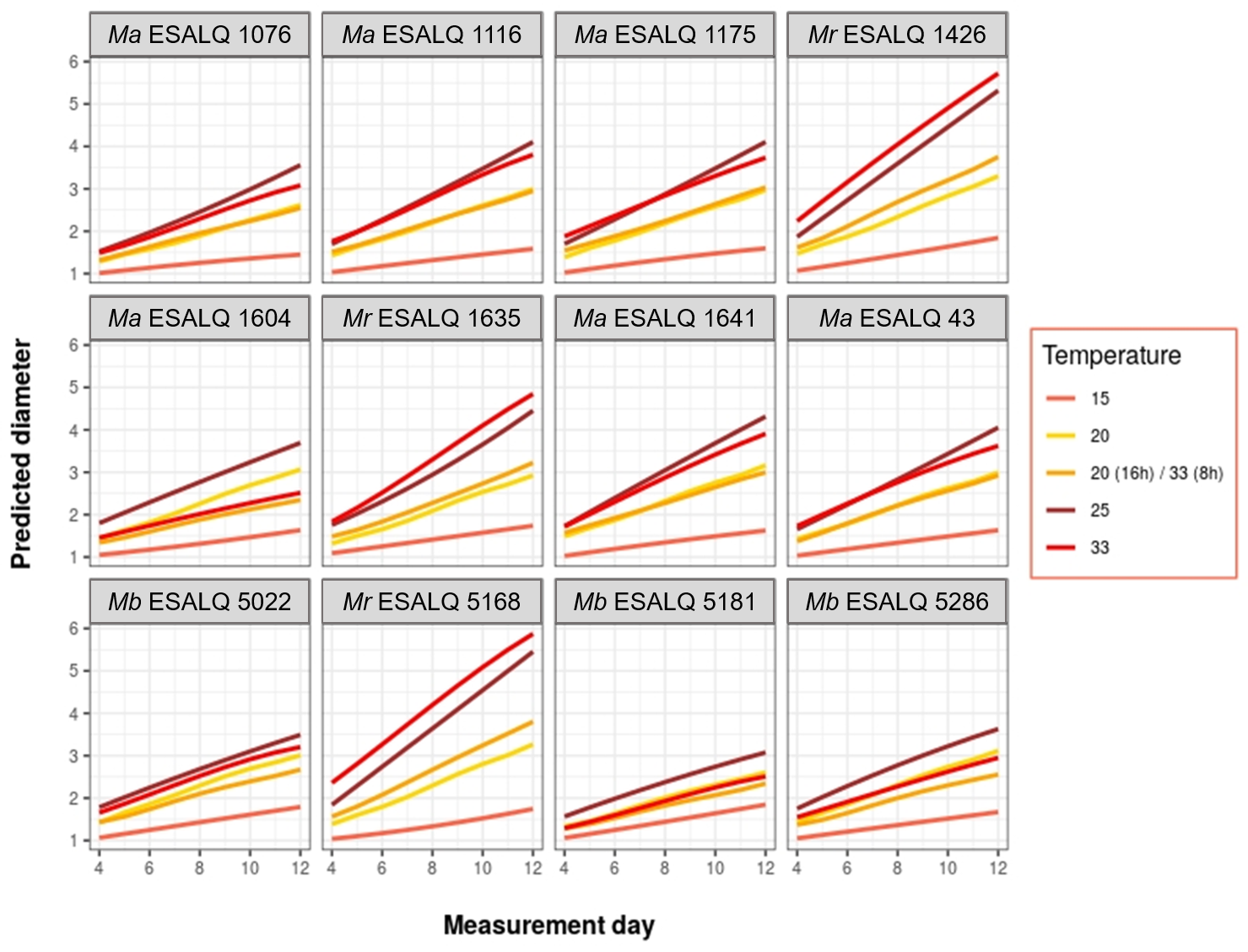

Supplement: Supplementary file 1 [file Data_Sheet_1.ZIP › Figure S2.tif]
